# Repeated Closed Head Injury in Mice Results in Sustained Motor and Memory Deficits and Chronic Cellular Changes

**DOI:** 10.1371/journal.pone.0159442

**Published:** 2016-07-18

**Authors:** Amanda N. Bolton Hall, Binoy Joseph, Jennifer M. Brelsfoard, Kathryn E. Saatman

**Affiliations:** 1 Spinal Cord and Brain Injury Research Center, University of Kentucky College of Medicine, Lexington, Kentucky, United States of America; 2 Department of Physiology, University of Kentucky College of Medicine, Lexington, Kentucky, United States of America; 3 Department of Neurosurgery, University of Kentucky College of Medicine, Lexington, Kentucky, United States of America; University of South Florida, UNITED STATES

## Abstract

Millions of mild traumatic brain injuries (TBIs) occur every year in the United States, with many people subject to multiple head injuries that can lead to chronic behavioral dysfunction. We previously reported that mild TBI induced using closed head injuries (CHI) repeated at 24h intervals produced more acute neuron death and glial reactivity than a single CHI, and increasing the length of time between injuries to 48h reduced the cumulative acute effects of repeated CHI. To determine whether repeated CHI is associated with behavioral dysfunction or persistent cellular damage, mice receiving either five CHI at 24h intervals, five CHI at 48h intervals, or five sham injuries at 24h intervals were evaluated across a 10 week period after injury. Animals with repeated CHI exhibited motor coordination and memory deficits, but not gait abnormalities when compared to sham animals. At 10wks post-injury, no notable neuron loss or glial reactivity was observed in the cortex, hippocampus, or corpus callosum. Argyrophilic axons were found in the pyramidal tract of some injured animals, but neither silver stain accumulation nor inflammatory responses in the injury groups were statistically different from the sham group in this region. However, argyrophilic axons, microgliosis and astrogliosis were significantly increased within the optic tract of injured animals. Repeated mild CHI also resulted in microgliosis and a loss of neurofilament protein 200 in the optic nerve. Lengthening the inter-injury interval from 24h to 48h did not effectively reduce these behavioral or cellular responses. These results suggest that repeated mild CHI results in persistent behavioral dysfunction and chronic pathological changes within the visual system, neither of which was significantly attenuated by lengthening the inter-injury interval from 24h to 48h.

## Introduction

An estimated 3.8 million people in the United States sustain a traumatic brain injury (TBI) every year, resulting in 60 billion dollars in annual healthcare costs [1,2]. Approximately 80% of TBIs are classified as “mild” head injuries [3–5], and often result in one or more symptoms such as nausea, dizziness, and/or headaches [4,6–8]. Memory loss, motor coordination deficits, vision impairment, anxiety, and/or irritability have also been observed after mild TBI [4,9]. Most mild TBI patients are discharged within 48h after their head injury [10,11], encouraged to rest and removed from activities that are a high risk for subsequent TBI until any and all symptoms have ceased [12]. However, the time necessary for all symptoms of mild TBI to subside can range from 1 week [6,8,13,14] to months [11] or even a year [15]. Varied responses to mild TBI and insufficient evidence correlating symptom presentation to cellular injury make it difficult to determine the brain’s period of vulnerability after a head injury. A repeated head injury occurring before the brain fully recovers can induce a phenomenon known as second impact syndrome causing hyperemia, intracranial hypertension and even death [16]. In less severe cases, individuals who have suffered two mild head injuries have been reported to perform worse on information processing tasks compared to individuals who sustained a single mild head injury [17]. Increasing evidence suggests that repeated mild TBI may be associated with changes in mood, cognition, and motor coordination over months to years and may develop into a condition now known as Chronic Traumatic Encephalopathy (CTE) [18,19]. However, the mechanism of injury and the role that repeated insults play in the development of pathology and behavioral consequences that associate with CTE is limited by (a) the sample size of confirmed human cases, (b) the accuracy of injury assessment and acquired behavioral histories from the patients and/or their families, and (c) the snapshot of disease progression at death.

Using animal models, insult severity can be modulated to create a single mild TBI as defined by minimal gliosis, axonal injury, and cell death and transient or mild motor or cognitive dysfunction. Numerous studies have shown that multiple injuries in close succession result in worsened behavioral function and/or histopathology compared to a single mild TBI [16,20–35]. Lengthening the inter-injury interval, or period of rest between injuries, typically limits brain damage or dysfunction following repeated mild TBI [21–24,33,35–38]. In our mouse model of mild TBI which impacts directly onto the intact skull, closed head injuries (CHI) repeated at 24h intervals produced increased acute neuronal death and axonal injury, enhanced astrogliosis, and induced microgliosis in several brain regions including the hippocampus, entorhinal cortex and cerebellum compared to single CHI or sham injury. These data are consistent with other studies of repeated mild TBI at 24h inter-injury intervals which report axonal injury [16,28], astrocytosis [32,38–41], and microgliosis [28,38,42] in the brain. Although similar pathological responses have been described with repeated mild TBI at 48h inter-injury intervals [30,43–45], 24h and 48h inter-injury intervals have not been previously compared within a single study, with the exception of our acute histopathology study. In our model, extending the inter-injury interval between CHI from 24h to 48h significantly reduced the amount of acute cell death and inflammation [23], supporting the hypothesis that a longer period of rest between head injuries allows the brain to recover and reduces the potential for exacerbation of the secondary injury cascade.

Both 24h and 48h inter-injury intervals have been separately reported to induce transient motor impairment [16,41,43] and persistent cognitive deficits for as long as a year after injury compared to sham injured mice [22,30], but have not been compared within the same study. We hypothesized that five repeated mild TBI at 24h inter-injury intervals would induce behavioral deficits and result in persistent inflammation and secondary injury out to 10wks. In addition, we anticipated that lengthening the inter-injury interval to 48h would lessen long-term behavioral deficits and neuropathology.

## Materials and Methods

### Animals

Two- to 3-month old male C57BL/6 mice were purchased from Jackson Laboratories (Bar Harbor, ME). Upon arrival, mice were group housed under a controlled 14:10h light:dark cycle and provided mouse chow and water *ad libitum*. Animal husbandry and all surgical procedures were approved by the University of Kentucky Institutional Animal Care and Use Committee and followed the federal guidelines set by the Institute of Laboratory Animal Resources (U.S.) and Committee on the Care and Use of Laboratory Animals.

### Repeated Closed Head Injury

Experimental CHI was induced following a previously described procedure [23]. Mice were anesthetized with 2.5% isofluorane delivered via a nose cone, the head of each mouse was fixed between two zygomatic bars stabilized in a stereotaxic frame. The incision site was first cleaned with 70% ethanol and betadine and local analgesia was achieved by subcutaneous injection of 0.2ml 1:200,000 epinephrine and 0.5% bupivacaine (Henry Schein Animal Health, Dublin, OH) in sterile, normal saline prior to scalp reflection. A pneumatically controlled cortical impact device (TBI-0310 Impactor, Precision Systems and Instrumentation, Fairfax Station, VA) with a 5mm diameter, cushioned tip of 55 Shore A hardness was programmed to deliver a 2.0mm impact at 3.5m/s with a 500ms dwell time. The posterior edge of the tip was aligned at the Lambda suture (approximately Bregma level -5mm). The diameter of the tip (5mm) is such that the anterior edge of the tip meets the Bregma suture (0mm Bregma level). Subsequent injuries were induced at the same location. This impact was previously characterized such that a single injury would result in minimal gliosis and cell death without resulting in skull fracture [23]. Immediately after impact, mice were removed from the stereotaxic device and placed onto their backs on a heating pad. Apnea duration and the time to spontaneously right to a prone position (righting reflex) were assessed. Upon righting, mice were briefly re-anesthetized to suture the scalp using vicryl sutures containing antibiotics (Ethicon, Cincinnati, OH). After suturing, 1ml of sterile saline was delivered subcutaneously to increase hydration after the injury. Sham-injured animals underwent identical anesthesia and surgical procedures without receiving an impact.

All mice were monitored on a heating pad until they became ambulatory. Additionally, mice were evaluated to 1-3h and 24h after each injury, followed by weekly inspections. Mice were rated to have no (0), mild (1), moderate (2), or severe (3) pain as indicated by locomotion in their home cage, pain on palpation of surgery site, abnormal behavior, and the appearance of the incision. Examples of pain responses included hunched posture, tenderness at the site of the incision, vocalizations, stumbling and/or hugging the cage. Humane endpoints were in place for mice scoring moderate or severe in at least one category, or mild in more than one category. In addition, all mice were required to maintain 85% of their starting weight in order to receive each subsequent head injury. However, no mice in the current study met either criterion for being removed from the study.

For all experiments, mice were randomized into three groups. The first injury group received five CHI, one every 24h (rCHI-24h; n = 10), whereas the second injury group received five CHI, one every 48h (rCHI-48h; n = 10). A third group consisted of sham CHI repeated every 24h for five days (sham; n = 10). We showed previously that sham-injured animals with anesthesia repeated at either 24h or 48h intervals were not significantly different from each other based on histology [23]; therefore, only one sham control group was utilized. One mouse assigned to the rCHI-24h group and one mouse assigned to the rCHI-48h group did not recover from their apneic episode after an injury, resulting in an n of 9 for each of these groups.

### Behavioral Analysis

#### Beam Walking

The beam walking test has been modified from our previously described protocol with the controlled cortical impact (CCI) model [46,47], which utilized four beams of varying widths to examine motor coordination after injury. Only the narrowest beam and rod were utilized in this study to enhance sensitivity as a milder deficit was anticipated. Prior to injury, mice were acclimated to beam walking using a 3cm width plexiglass beam. Twenty-four hours after the final injury or sham procedure, mice were scored on their ability to walk across a 0.5cm width, plexiglass beam and a 0.5cm diameter, wooden dowel rod. Mice received 3pts for walking across the beam without having a foot slip or inverting on the beam. One point was deducted if one or more foot slips occurred, an additional point was deducted if the animal inverted under the beam, and a score of zero was given if the mouse fell off the beam or could not cross. On the rod, a maximum score of 2pts was given for walking across the rod; a point was deducted if mice inverted more than two times while crossing, and a score of zero was given if the mouse fell off the rod or could not cross. These tasks were repeated on the following testing days: 3d, 10d, 17d, 24d, 6wk, 8wk, and 10wk. For correlations with histological quantifications, the average beam walking score across the 10wk period was used.

#### Novel Object Recognition

The novel object recognition (NOR) test has been previously established in our lab and others to test cognition after TBI in rodents [46–48]. Prior to injury, mice were acclimated to a 10.5” x 19” x 8” plastic box for one hour. Eight days after the final injury or sham procedure, mice were placed back into their individual testing box and allowed to explore two identical objects placed in opposite corners for five minutes. Four hours later, mice were placed back into their testing cage for five minutes with one of the previously explored objects and a novel object. The amount of time spent exploring each object was recorded. Additional time in the testing box was allowed to ensure a total object exploration time of at least ten seconds. Three mice (two rCHI-48h, one rSHAM) required an extra 1–3 minutes on the first testing day. Recognition Index was calculated as the time spent exploring the novel object divided by the combined familiar and novel object exploration times, and was expressed as a percentage. For each additional testing day at 2wk, 4wk, 8wk, and 10wk post-injury, mice were returned to their testing cage for five minutes to explore a distinct novel object and the same familiar object.

#### Gait Analysis

On the third day following the final injury, mice were placed in a Digigait box with a clear treadmill belt (Mouse Specifics, Inc., Framingham, MA). A camera positioned underneath the belt recorded the ventral aspect of the mouse. The belt was set to 15cm/s, a speed which required the animals to continuously walk. Five consecutive seconds of video in which the mouse moved within the same frame were utilized for analysis. This task was repeated at 1mo after injury. The videos were then analyzed with the Mouse Specifics software using established protocols to measure gait and paw placement [49,50].

### Tissue Processing

Following the final behavioral test, mice were euthanized by intraperitoneal injection of Fatal Plus (130mg/kg, Henry-Schein Animal Health, Dublin, OH) before transcardial perfusion with cold, heparinized sterile saline followed by cold, 4% paraformaldehyde (PFA) for ten minutes. After perfusion, mice were decapitated and the heads placed into vials of 4% PFA for 24h. The brains and optic nerves were then removed from the skull and post-fixed in 4% PFA for an additional 24h. Following post-fixation, tissue was placed into 30% sucrose in 1X-Tris-buffered saline (TBS) for 48h for cryoprotection. The brain tissue was frozen in -25 to -35°C isopentane before being cut into 40μm thick coronal sections using a sliding microtome (Dolby-Jamison, Pottstown, PA). For optic nerves, tissue was frozen in optimal cutting temperature compound (OCT) on the sliding microtome and cut into 10μm thick longitudinal sections. Tissue sections were stored at -20°C in 30% glycerol, 30% ethylene glycol in 1X TBS.

### Histopathological Analysis

#### Histology

Silver staining was performed on a series of brain tissue which included 12 sections spaced at 400μm for the cerebrum and 200μm for the cerebellum/brainstem. The FD NeuroSilver kit (FD NeuroTechnologies, Ellicott City, MD) was used with the following two modifications from the manufacturer’s instructions: (1) for the step involving the mixture of solution C and F, tissue was placed into the solution 2x 2.5 minutes; (2) tissue was dehydrated sequentially in 70%, 80%, 95%, and 100% ETOH, and cleared in Xylenes prior to coverslipping.

#### Immunohistochemistry

A series of free-floating tissue sections spaced at 400μm apart were used for immunohistochemistry. For procedures utilizing 3,3’-diaminobenzidine, tissue sections were treated with 3% H_2_O_2_ in 50/50 methanol/ddH_2_O for 30 minutes in order to quench endogenous peroxides. For all immunohistochemical protocols, tissues were blocked with 5% normal horse serum in 0.1%Trition X-100/1XTBS before incubation in primary antibody (Table 1) overnight at 4°C. On the following day tissue sections were rinsed and incubated in the appropriate secondary antibody (Table 1) for 1 hour. For Iba-1 labeled tissue, the tissue was washed after incubation in Alexa 488-conjugated secondary antibody, mounted onto gelatin-coated slides, and coverslipped using Fluoromount mounting media (Southern Biotech, Birmingham, AL). For all other protocols using biotinylated secondary antibodies, the tissue was washed before incubating in Avidin-Biotin complex (Vector Laboratories, Burlingame, CA) for 1 hour and then treating with 3,3’-diaminobenzidine as directed by the manufacturer (Vector Laboratories).

**Table 1:** Antibody List.

Antibody Name	Public Identifier	Supplier	Cat #*; Log #*	Clone; Host	Immunogen	Concentration	Validation study
Anti-ionized calcium-binding adaptor molecule 1	Iba-1	Wako; Richmond, VA	019–19741*; CTR-6026*	Poly; Rabbit	Synthetic peptide corresponding to C-terminus of Iba-1	1:1000	[51]
Anti-glial fibrillary acidic protein	GFAP	Sigma-Aldrich; St. Louis, MO	G9269*; 127K4807*	Poly; Rabbit	GFAP from human brain	1:1000	[52]
Anti-cluster of differentiation-68,	CD68	Bio-Rad; Herclues, CA	MCA1957*; 0114*	Mono clone FA-11; Rat	Purified concanavalin A acceptor glycoprotein from P815 cell line.	1:1000 (cerebrum); 1:500 (optic nerve)	[53]
Anti-neurofilament protein-200	NF200	Sigma-Aldrich; St. Louis, MO	N0142*; 017K4802*	Mono; Mouse	C-terminal segment of enzymatically dephosphorylated pig neurofilament 200	1:100	[54]
Anit-paired helical filament-1	PHF-1	The Feinstein Institute Great Neck, NY	n/a	Mono; Mouse	Soluble PHF	1:500	[55]
Donkey anti-rabbit IgG, Biotin-SP conjugate	Dk anti Rb IgG Biotin	Jackson Immuno; West Grove, PA	711-065-152*; 11372*	Mono; Mouse	Whole IgG	1:1000	—
Donkey anti-rat IgG, Biotin-SP conjugate	Dk anti Rt IgG Biotin	Jackson Immuno; West Grove, PA	712-065-153*; 106342*	Poly; Donkey	Whole IgG	1:1000	—
Donkey anti-mouse IgG, Biotin-SP conjugate	Dk anti Ms IgG biotin	Jackson Immuno; West Grove, PA	715-065-151*; 107570*	Poly; Donkey	Whole IgG	1:1000 (cerebrum); 1:500 (optic nerve)	—
Donkey anti-rabbit IgG, Alexa Fluor 488, conjugate	Dk anti Rb Alexa 488	Life Technologies; Carlsbad, CA	A21206*; 1275888*	Poly; Donkey	Gamma immunoglobins heavy and light chains	1:2000	—

#### Quantification of Histology

All analyses were performed by an examiner blinded to the injury conditions of each animal.

In our previous study, inflammation was increased 24h following repeated mild TBI at 24h inter-injury intervals in the entorhinal cortex and hippocampus [23]. Therefore, GFAP immunoreactivity was analyzed at 10wks post-injury in the entorhinal cortices and hippocampi (4 sections/animal) and Iba-1 immunoreactivity was analyzed in the entorhinal cortices (4 sections/animal) as previously described [23]. In brief, GFAP and Iba-1 images were taken using an Olympus BX51 microscope with an ASI XY automated stage, and a montage of the images were created using Image Pro Plus software. For GFAP immunolabeling, the mean integrated optical density (IOD) of was measured and averaged across sections for statistical analyses. For Iba-1 labeling, an examiner chose the display range (between 0 and 255) that selected immunoreactive microglia for each tiled image. The percent area of Iba-1 immunoreactivity was measured and averaged for statistical analyses.

Silver-stained sections were viewed at 10x magnification using an Olympus BX51 microscope with an ASI XY automated stage. Images of the tissue were captured with the calibrated Stage-Pro module of Image Pro Plus (Media Cybernetics, MD) and assembled into a montage. Based on initial light microscopic evaluation of the cerebrum and brainstem, regions with notable positive staining were selected for quantitative analysis. In the cerebrum, the optic tract and peduncle were analyzed bilaterally in 2–3 sections/animal. In the brainstem, the pyramidal tract and adjacent grey matter were analyzed in two sections/animal. Each area of interest (AOI) was outlined using the anatomical markers found in “The Mouse Brain in Stereotaxic Coordinates”, 4^th^ Edition (Paxinos & Franklin). Utilizing the entire range of pixel densities (0–255), the mean density of each AOI was recorded. For each animal, the mean density was averaged across sections for each anatomical area for statistical analyses. Silver staining in the cerebellar lobes was evaluated qualitatively.

Following the observations in the optic and pyramidal tracts with silver stain, inflammation was also evaluated in these regions using immunohistochemical labeling of CD68 and GFAP for activated microglia or astrocytosis, respectively. For analysis, tissue sections were imaged using the Olympus BX51 microscope (10x) with a Q Imaging camera. Each AOI was outlined as described above. An examiner then chose the density range that selected immunoreactive microglia or astrocytes for each image. The percent of the total area which contained CD68 labeled microglia or GFAP labeled astrocytes were measured and averaged bilaterally for the optic tract across 2–3 sections/animal and for the pyramidal tract in 1 section/animal.

Neurodegeneration observed in the visual pathway prompted an evaluation of the optic nerves for axon loss and ongoing microgliosis. NF200 immunolabeling was used to visualize intact axons and CD68 immunolabeling was used to examine activated microglia. For quantitative analysis, each nerve was imaged using an Olympus AX80 microscope (10x) and a DP-70 camera. Several overlapping images were obtained to encompass the length of the optic nerve from the retina to the optic chiasm. These images were used to create a montage of the whole nerve in Adobe Illustrator CS6. The intensity threshold that detected labeled neurofilament protein or activated microglia was selected. The area of positive labeling was normalized to the AOI for statistical analyses.

PHF-1 labeled tissue was evaluated qualitatively. Brain tissue from a 3.5mo old rTg4510 tau mouse was used as a positive control for PHF-1 labeling [56].

### Statistics

Analyses were completed using Graph Pad Prism 5 or Statistica 5.0 software. For apnea, righting reflex, and behavioral tests, a repeated measures 1-way ANOVA (time x injury group) was performed followed by post-hoc Newman-Keuls tests where appropriate. For each histological marker within each region, a 1-way ANOVA followed by post-hoc Newman-Keuls tests where appropriate was performed. For correlations between histological and behavioral outcomes, Spearman’s correlation was performed, and Spearman’s rho and corresponding p values are reported. All data are presented as means with standard error.

## Results

### Apnea and Righting Reflex

As in our previous report using this model of mild TBI [23], both apnea duration and time to right increased in length after CHI and were maximal after the first injury, decreasing in duration with subsequent impacts (Table 2). For apnea duration there was a significant main effect of injury group (p<0.05) and CHI number (p<0.05) but the interaction between the two was not statistically significant (p = 0.07). Post-hoc comparisons among groups indicated that rCHI at either 24h or 48h increased apnea compared to sham injury ($ indicates p<0.05 compared to sham). In addition, post-hoc analysis for the main effect of CHI number demonstrated that apnea duration was highest after CHI 1 and CHI 2 (# indicates p<0.05 compared to CHI 3, 4, and 5). Time to right after injury was significantly dependent on the injury group (p<0.05) and the CHI number (p<0.05; interaction p<0.05). Post-hoc analyses revealed a significant increase in the time to right after the first CHI (p<0.05 when compared to sham injury) and a trend toward an increase after the second impact in the rCHI-24h group (p = 0.08). Apnea duration and righting reflex were not significantly different between animals in the rCHI-24h and rCHI-48h groups. At this impact depth, CHI did not produce skull fractures in any mice.

**Table 2:** Apnea and Righting Reflex.

	CHI 1		CHI 2		CHI 3	CHI 4	CHI 5
	#		#				
Apnea (sec)							
rSHAM	0		0		0	0	0
rCHI-24h	26 ± 7		21 ± 7		15 ± 6	8 ± 4	10 ± 2
rCHI-48h	22 ± 4		21 ± 5		8 ± 3	11 ± 3	5 ± 2
Righting							
rSHAM	1:15 ± 0:04		1:11 ± 0:06		1:13 ± 0:05	1:21 ± 0:06	1:26 ±0:05
Reflex (min)							
rCHI-24h	9:13 ± 2:46	$	6:03 ± 2:04	p = 0.08	2:33 ± 0:29	1:49 ± 0:15	2:15 ± 0:15
rCHI-48h	10:40 ± 2:28	$	5:35 ± 1:50		2:08 ± 0:28	2:05 ± 0:15	1:46 ± 0:11

Apnea and the time to flip to prone position (righting reflex) were recorded after each of five closed head injuries.

^$^ indicates p<0.05 compared to sham.

^#^ indicates p<0.05 compared to CHI 3, 4, and 5.

### rCHI Induces Persistent Memory Dysfunction

**Fig 1:**
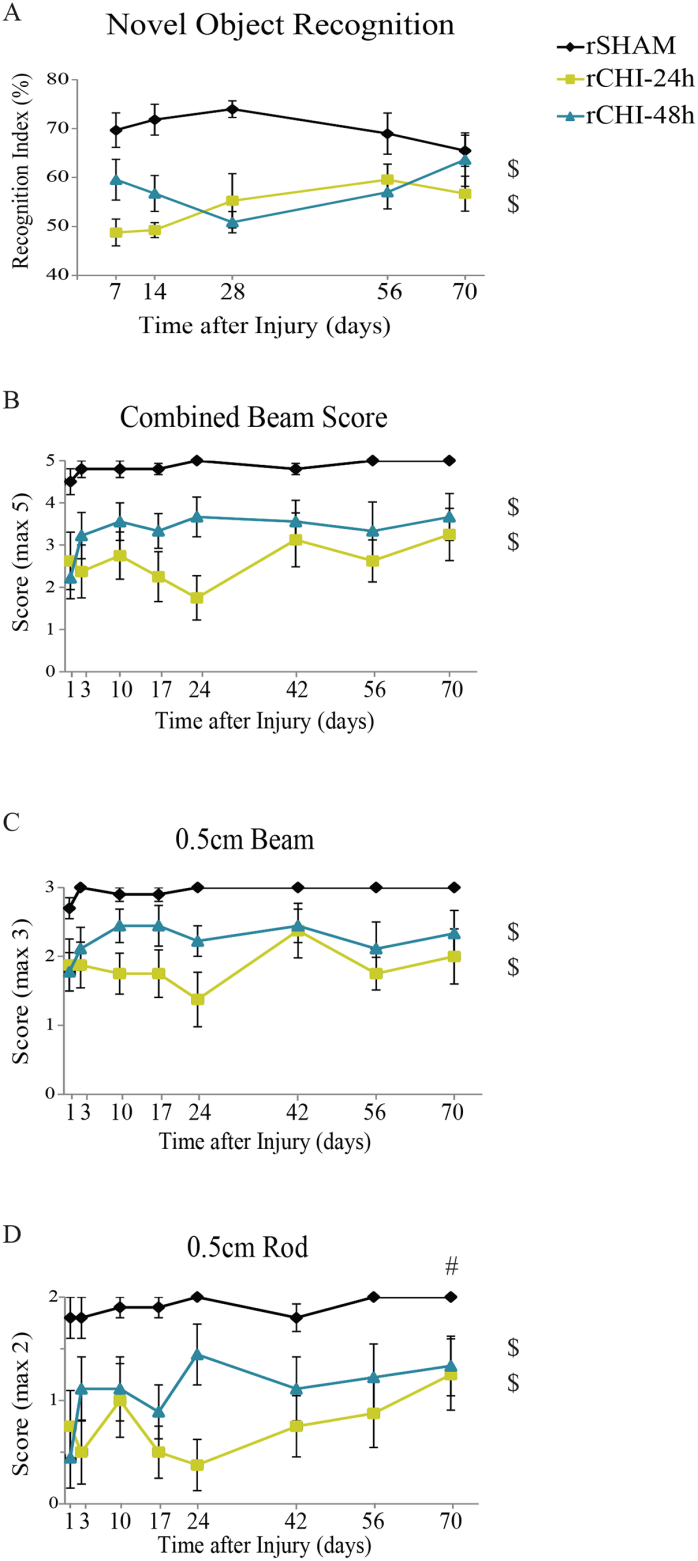
Repeated closed head injury (CHI) induced motor and memory deficits over 10wks post-injury. Behavioral testing was conducted following repeated sham (rSHAM), repeated CHI at a 24h interval (rCHI-24h), and repeated CHI at a 48h interval (rCHI-48h). (A) Memory scores in the novel object recognition task were calculated by dividing the time spent exploring the novel object by the total exploration time (recognition index). (B) The beam walking task was used to identify deficits in motor coordination. A score of 5 indicated perfect performance on the task, with lower scores indicating poorer motor skills. Component analysis for the beam walking task of (C) the 0.5cm plexiglass beam and (D) the 0.5cm wooden dowel rod. $ indicates significant difference from rSHAM (p<0.05 post-hoc testing for main effect of injury). # indicates that performance across all groups was better at 10wks compared to 24h (p<0.05).

The novel object recognition (NOR) task was used to compare memory function among the rSHAM, rCHI-24h, and rCHI-48h groups at several time points across 10wks (Fig 1A). In our hands, naïve and sham-injured mice spend about 70–75% of total exploration time on the novel object [47]. Mice receiving repeated sham injury exhibited this same behavior with a recognition index of approximately 70%. Memory ability was dependent upon injury status (p<0.05) but not time after injury (p>0.05; Interaction, p>0.05). CHI repeated at either 24h intervals or 48h intervals induced a persistent deficit in cognition across the testing period (p<0.05 compared to rSHAM). Lengthening the inter-injury interval from 24h to 48h appeared to result in a milder initial cognitive deficit, but this difference was not sustained across the 10wk period (p = 0.1).

### rCHI Induces Deficits in Motor Coordination with Beam Walking but not Gait Analysis

Following the final day of injury, mice were assessed on a beam walking task at several time points to examine deficits in motor coordination after rCHI (Fig 1B). Beam walking scores were dependent upon the injury group (p<0.05) but not the amount of time after injury (p>0.05; Interaction p>0.05). Post-hoc testing among injury groups revealed that CHI repeated at either 24h intervals (p<0.05) or 48h intervals (p<0.05) produced significant motor deficits compared to sham injury. Although rCHI-24h appeared to result in a larger beam walking deficit than rCHI-48h across 10wks, this did not reach statistical significance (p = 0.1). To determine whether combining the beam and rod portions of the test masked subtle deficits better detected by one task, scores were analyzed separately (Fig 1C and 1D). Individual analyses of the beam and rod corroborated the combined beam test, with rCHI at 24h and 48h intervals inducing significant motor deficits compared with sham injury. However, performance on the rod varied as a function of time, with significant recovery at 10wks compared with 24h post-injury (Fig 1D). To determine whether the motor deficit observed in rCHI animals might have been a result of the first impact and not a cumulative effect, we analyzed beam walking data from our previous acute CHI study [23] in which mice were evaluated 2h and 24h after each impact. Two hours after a single CHI, mice (n = 15) scored an average of 1.8 ± 0.2 on the 0.5cm beam (maximum score of 3) and 1.8 ± 0.1 on the rod (maximum score of 2). By 24h after a single CHI, beam walking scores (2.8 ± 0.1 on the beam and 2.0 ± 0.0 on the rod) were comparable to those of sham mice, suggesting a single CHI resulted in only mild transient motor dysfunction which resolved by 24h.

Gait coordination was assessed using a treadmill test (Digigait). Mice were tested three days after the final injury, as well as at 1 month after injury. A large number of parameters can be examined using the Digigait software. Results from analyses of gait symmetry, hindlimb shared stance, and paw area at peak stance are provided in Table 3. No significant differences were observed among groups in any parameter at either of the time points after injury.

**Table 3:** Digigait Analysis.

		rSHAM		rCHI-24h		rCHI-48h	
Timepoint:		3d	1mo	3d	1mo	3d	1mo
Gait Symmetry		0.90 ± 0.05	0.95 ± 0.07	0.97 ± 0.06	0.94 ± 0.05	0.97 ± 0.06	0.92 ± 0.05
Hindlimb Shared Stance (sec)		0.13 ± 0.02	0.14 ± 0.02	0.13 ± 0.02	0.15 ±0.03	0.12 ± 0.02	0.13 ± 0.03
Paw Area at Peak	Fore:	0.31 ± 0.02	0.28 ± 0.03	0.28 ± 0.03	0.26 ± 0.02	0.29 ± 0.03	0.26 ± 0.04
Stance (cm^2^)	Hind:	0.56 ± 0.07	0.52 ± 0.08	0.55 ± 0.07	0.47 ± 0.08	0.56 ± 0.08	0.52 ± 0.12

### Chronic Neurodegeneration after Mild TBI Irrespective of Inter-injury Interval

**Fig 2:**
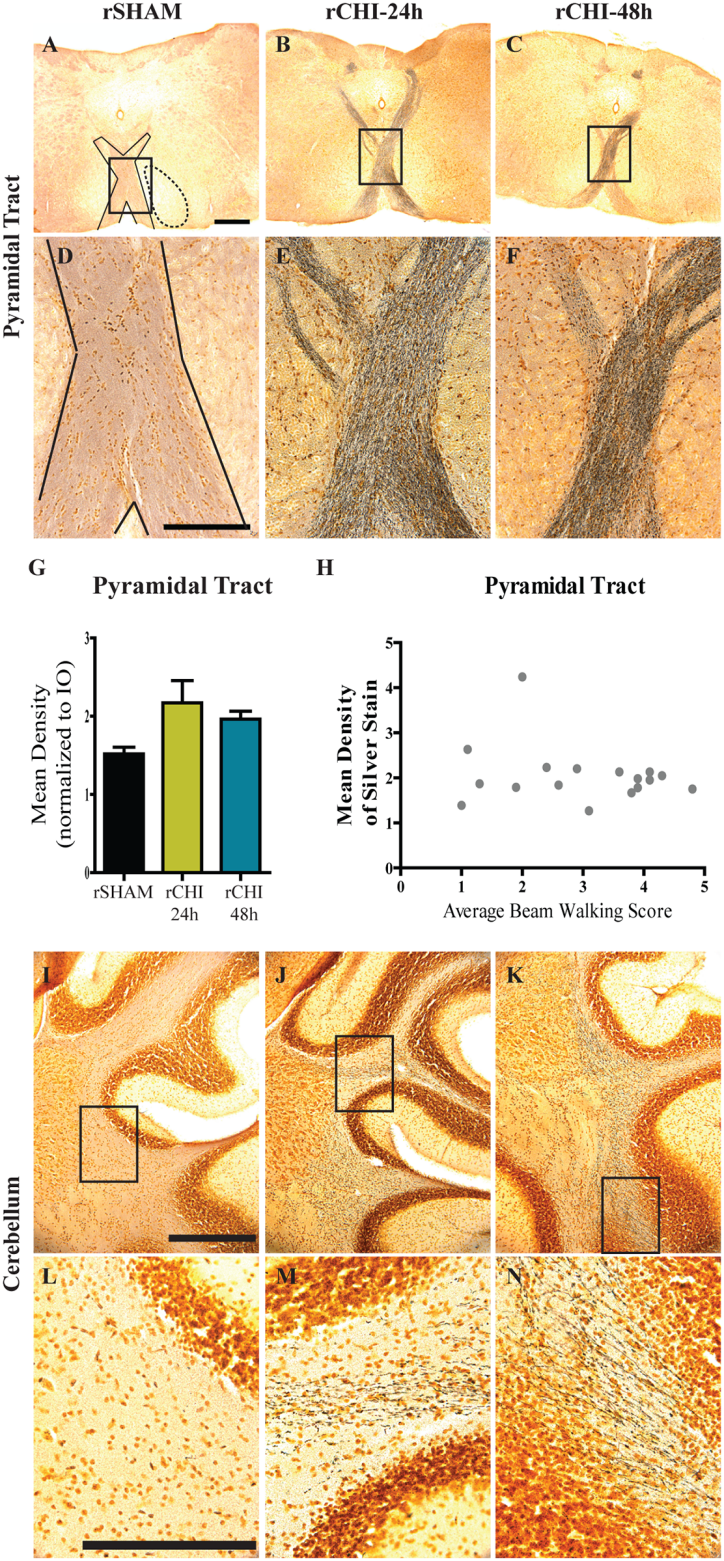
Repeated closed head injury (CHI) induced chronic axonal degeneration in the pyramidal tract and cerebellar crusiforms. Argyrophilic axons in the pyramidal tract (A-F) and cerebellar crusiforms (I-N) were labeled using silver stain. Scale bars: 500μm A-C, I-K; 250μm D-F, L-N). The mean density of silver staining in the pyramidal tract (G) was normalized to background using adjacent stained areas. Correlation between pyramidal tract silver stain after repeated CHI and average beam walking score across 10wks (H).

Conventional Hematoxylin and Eosin stain did not reveal contusion, hemorrhage or overt neuron death or atrophy (S1 Fig). Silver stain was used to label neurons undergoing degeneration [57] and has been used previously to demonstrate axonal injury after closed head injury [28,29,58]. In our hands, the staining yielded a copper-toned background with black particulates in the degenerating regions. No evidence of neurodegeneration in the neocortex, hippocampus, or corpus callosum was observed after repeated CHI or sham injury. However, repeated CHI induced bilateral degeneration in the pyramidal tract of the brainstem (Fig 2B, 2C, 2E and 2F) and in the white matter regions of the cerebellar crusiforms (Fig 2J, 2K, 2M and 2N). The mean density of silver stain in the pyramidal tract (Fig 2A, solid outline) was normalized to the adjacent inferior olive (Fig 2A, dotted outline). While some animals (7 of 9 in rCHI-24h and 4 of 9 in rCHI-48h) exhibited pyramidal tract degeneration after repeated CHI, this effect did not reach statistical significance (ANOVA p = 0.052; Fig 2G). As the pyramidal tract contains corticospinal axons involved in motor control, we postulated that persistent motor deficits might be related to the degree of pyramidal tract degeneration. However, the density of pyramidal tract silver staining in injured animals did not significantly correlate with their average beam walking score (p>0.05; r = 0.12; Fig 2H).

**Fig 3:**
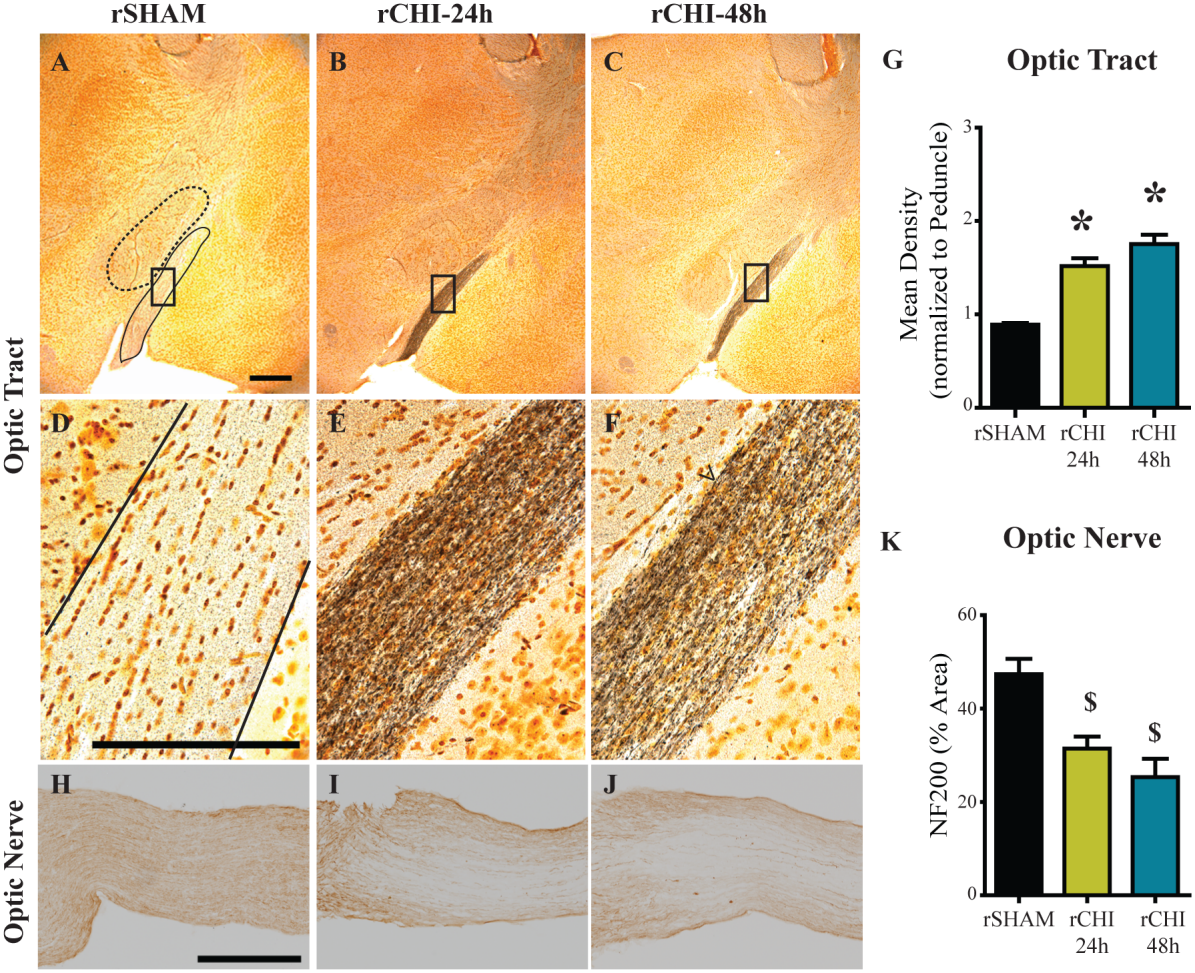
Repeated closed head injury (CHI) induced chronic axonal degeneration in the optic tract and optic nerve. Argyrophilic axons in the optic tract (A-F) were labeled using silver stain. Scale bars: 500μm A-C: 250μm D-F. The mean density of silver staining in the optic nerve was normalized to background using adjacent stained areas (G). * indicates p<0.05 compared to all other groups. Optic nerves (H-J) were labeled for neurofilament heavy chain (NF200) and the percent area of staining was quantified (K). Scale bars: 250μm H-J. $ indicates p<0.05 compared to sham.

In the cerebrum, axons within the optic tract were positively labeled with silver stain in repeated CHI (Fig 3B, 3C, 3E and 3F) but not sham-injured (Fig 3A and 3D) mice. The mean density of silver stain within the optic tract (Fig 3A, solid outline) was normalized to the thalamic peduncle adjacent to the optic tract (Fig 3A, dotted outline) to control for variation in background intensity across animals. Silver stain mean density in the optic tract of the rCHI-24h group (p<0.05) and rCHI-48h group (p<0.05) was significantly increased compared to rSHAM (Fig 3G). Optic tract silver staining was greater in the rCHI-48h group than the rCHI-24h group (p<0.05). Neurodegeneration within the optic tracts as well as a recent study published by Tzekov and colleagues [45] prompted us to evaluate the optic nerves of brain-injured animals for additional damage. In an uninjured or sham animal, axons stained positively for NF200, the heavy chain component of neurofilaments (Fig 3H). Damaged optic nerves, in contrast, exhibited loss of NF200 labeling (Fig 3I and 3J), which was statistically significant for CHI repeated at either 24h or 48h intervals (p<0.05 compared to sham; Fig 3K). The interval for induction of CHI did not significantly influence the loss of NF200 immunolabeling (p>0.05).

### Persistent Inflammation after Mild TBI in Optic Tract and Pyramidal Tract but not Entorhinal Cortex and Hippocampus

Microglia and astrocyte reactivity were measured in several brain regions by Iba-1/CD68 immunoreactivity and GFAP immunoreactivity, respectively. Iba-1 labels all microglia. However, morphological features were used to delineate ‘activated’ microglia from ‘unactivated’ or resting microglia. In contrast, CD68 selectively labels activated microglia which appeared with swollen cell bodies and thick processes.

**Fig 4:**
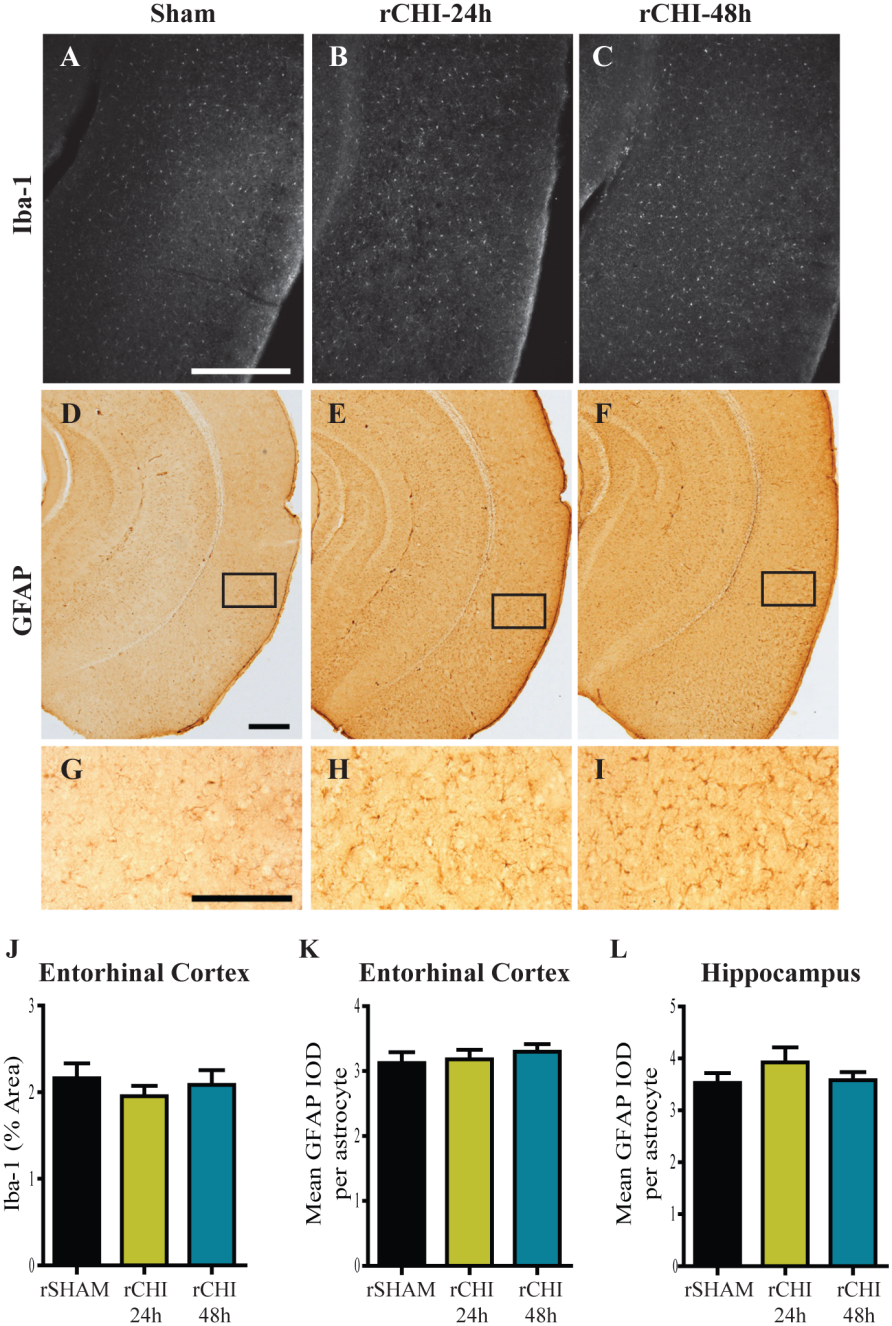
Inflammation did not persist in the entorhinal cortex or hippocampus 10wks after repeated closed head injury (CHI). Immunohistochemical labeling of ionized calcium-binding adaptor protein-1 (Iba-1; A-C) and glial fibrillary acidic protein (GFAP; D-I) in the entorhinal cortex and hippocampus after repeated sham (rSHAM), repeated CHI at 24h intervals (rCHI-24h), and repeated CHI at 48h intervals (rCHI-48h). Scale bars: 500μm A-F; 250μm G-I. The percent area of Iba-1 labeling was quantified for analysis in the entorhinal cortex (J). The mean integrated optical density (IOD) of GFAP/astrocyte was analyzed for the entorhinal cortex (K) and the hippocampus (L).

Due to acute microgliosis and astrocyte activation previously noted in the entorhinal cortex and hippocampus 24h following repeated CHI [23], these regions were analyzed at 10wks post-injury. In the entorhinal cortex, only a few hypertrophic microglia were observed at 10wks post-injury. The vast majority of Iba-1 labeled microglia had a resting morphology with small cell bodies and thin processes (Fig 4A–4C). The area of Iba-1 labeled microglia relative to a defined area of interest was comparable across all groups (p>0.05; Fig 4J). Iba-1 labeled microglia in the hippocampi were observed qualitatively and no differences were observed among groups. GFAP-positive astrocyte cell bodies and processes in the entorhinal cortex and hippocampus of rCHI animals did not appear swollen or increased in number compared to those in rSHAM animals (Fig 4D–4I). When quantified, the mean GFAP IOD/astrocyte in the entorhinal cortex was comparable across all groups (p>0.05; Fig 4K). In the hippocampus, one animal in the rCHI-24h group had the highest mean IOD/astrocyte of 5.9. However, on average the groups were comparable to each other (p>0.05; Fig 4L).

**Fig 5:**
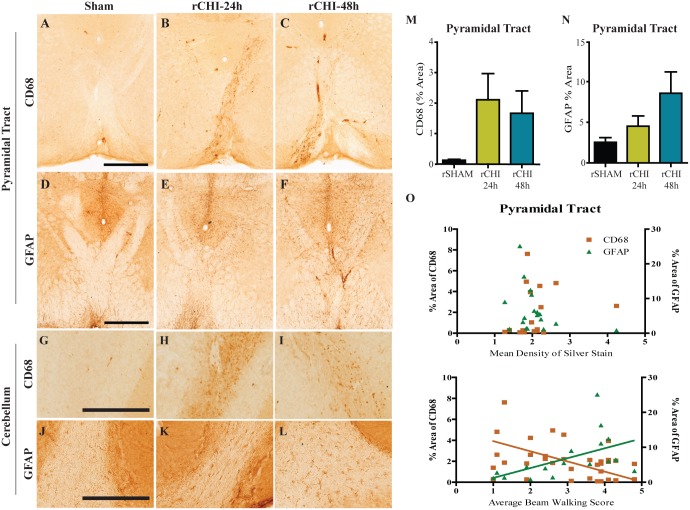
Repeated closed head injury (CHI) causes chronic activation of microglia and reactive astrocytes in the cerebellum and brainstem. Immunohistochemical labeling of cluster of differentiation 68 (CD68) and of glial fibrillary acidic protein (GFAP) in the pyramidal tract (A-C and D-F, respectively) and white matter tracts of cerebellar crusiforms (G-I and J-L, respectively) after repeated sham (rSHAM), repeated CHI at 24h intervals (rCHI-24h), and repeated CHI at 48h intervals (rCHI-48h). Scale bars: 500μm A-F; 250μm G-L. The percent area of CD68 and GFAP labeling was quantified for analysis in the pyramidal tract (M and N, respectively). The percent area of CD68 (orange squares) and GFAP (green triangles) labeling in the pyramidal tract did not correlate with the mean density of silver stain in the pyramidal tract (O top panel). The percent area of CD68 or GFAP labeling after repeated CHI compared to the average beam walking score across 10wks (O bottom panel). Lines represent linear regression for visualization purposes.

In the pyramidal tract (Fig 5A) and cerebellar crusiforms (Fig 5G) of rSHAM animals very few, if any, CD68-postive microglia were observed. Repeated CHI resulted in an appreciable increase in activated microglia in the pyramidal tract (Fig 5B and 5C) and cerebellum (Fig 5H and 5I), in a subset of animals injured at 24h (5 of 9) or 48h (3 of 9) intervals. However, across the entire group, the percent area of CD68 was not significantly increased within the pyramidal tract of rCHI animals compared to rSHAM animals (ANOVA p>0.05, Fig 5M). A mild astrocytic response was observed within the pyramidal tract (Fig 5E and 5F) and cerebellar crusiforms (Fig 5K and 5L) of injured animals. While a few animals from each injury group had a higher percent area of GFAP labeling in the pyramidal tract compared to sham animals, groups were not significantly different from each other (p>0.05; Fig 5N). Because neuroinflammation is often concomitant with neurodegeneration [23,40] we asked whether pyramidal tract degeneration predicted the extent of gliosis. Neither microgliosis nor astrocytosis correlated with increases in silver stain (p>0.05; r = 0.33 for silver stain v. CD68; r = -0.37 for silver stain v. GFAP; Fig 5O). Surprisingly, GFAP immunoreactivity in the pyramidal tract correlated with better average beam walking scores in injured animals (p<0.05; r = 0.67; Fig 5O) while, CD68 immunolabeling appeared to be inversely correlated with beam walking scores, although the correlation did not reach statistical significance (p = 0.056; r = -0.47; Fig 5O).

**Fig 6:**
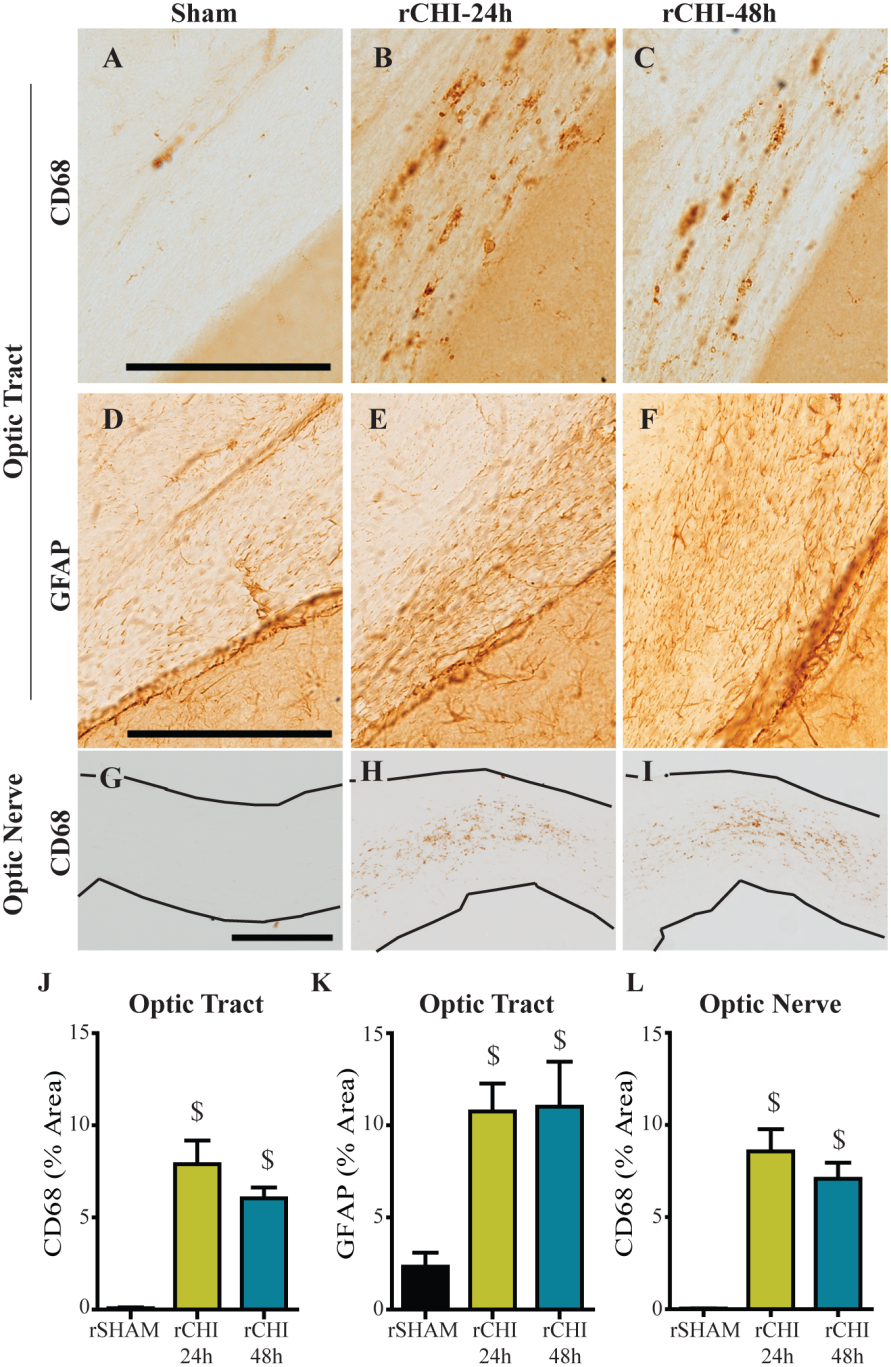
Repeated closed head injury (CHI) causes chronic microgliosis in the optic tract and optic nerve. Immunohistochemical labeling of cluster of differentiation 68 (CD68; A-C, G-I) and glial fibrillary acidic protein (GFAP; D-F) after repeated sham (rSHAM), repeated CHI at 24h intervals (rCHI-24h), and repeated CHI at 48h intervals (rCHI-48h). Scale bars: 250μm. The percent area of CD68 labeling was quantified for analysis in the optic tract (J), and optic nerves (L). The percent area of GFAP labeling was quantified for analysis in the optic tract (K). $ indicates p<0.05 compared to sham.

In the optic tract of injured animals, CD68-positive microglia were diffusely distributed (Fig 6B and 6C). In addition, GFAP-labeled astrocytes appeared to increase in number and were enlarged throughout the region (Fig 6E and 6F). The percent area of CD68 labeling (Fig 6J) and of GFAP-labeled astrocytes (Fig 6K) were significantly increased 10wks after repeated CHI at either 24h or 48h inter-injury intervals (p<0.05 compared to sham). CD68-positive microglia were also observed throughout the optic nerves of injured animals (Fig 6H and 6I), congregated most heavily in the areas where NF200 labeling was diminished. The percent area of CD68 labeling was significantly increased 10wks after repeated CHI at either 24h or 48h inter-injury intervals compared to sham injury (p<0.05; Fig 6L). Increasing the inter-injury interval from 24h to 48h did not significantly reduce gliosis in the optic tract or optic nerve 10wks after injury (p>0.05).

To determine whether microgliosis in the optic tract was initiated early after rCHI or represented a delayed response to ongoing neurodegeneration, we evaluated archival tissue from our previous study with this model [23]. Groups included repeated CHI (rCHI-24h (n = 7) and rCHI-48h (n = 8)) euthanized 24h after the final injury, single CHI (euthanized at 24h (n = 8), 5d (n = 5), or 9d (n = 5) post-injury) and repeated sham (n = 8). CD68 immunolabeling in the optic tract 24h after a single CHI was similar to labeling in rSHAM animals (p>0.05; S2 Fig). However, by five days after a single CHI, CD68 immunolabeling in the optic tract was significantly increased (p<0.05 compared to rSHAM). Microglial reactivity decreased significantly by nine days after a single CHI compared to five days after a single CHI (p<0.05) but remained elevated compared to rSHAM (p<0.05). When CHI were repeated at a 24h or 48h inter-injury interval, the percent area of CD68 labeling was significantly increased compared to that in rSHAM animals (p<0.05), but the response was similar to the peak microglial response seen five days after a single CHI.

### Absence of Pathological Tau

Neuronal inclusions of hyper-phosphorylated tau are a hallmark of CTE in humans, a condition associated with repeated TBI. In the current study mice with repeated CHI did not exhibit hyper-phosphorylated tau when immunolabeled with PHF-1 antibody 10wks after injury (S3 Fig). Transgenic mice modified to express human tau (rTg4510) exhibit age-related tau hyerphosphorylation [56]. PHF-1 accumulation was detected in brain sections from a 3.5 month old rTg4510 mouse, serving as a positive control for the protocol.

## Discussion

We previously established a model of mild TBI in which the extent of acute histopathology was dependent on injury severity and was amplified by repeated impacts if the inter-injury interval was 24h but not 48h [23]. Using this same model, five CHI resulted in persistent cognitive and motor dysfunction over a 10wk period as well as neurodegeneration and neuroinflammation in the visual pathway, the corticospinal tract and the cerebellum. These injury-induced changes were not effectively mitigated by extending the inter-injury interval from 24h to 48h.

### Motor Pathway Damage

Motor dysfunction is not a prominent feature of mild TBI, but difficulties in motor coordination are a common symptom associated with CTE. A number of repeated mild TBI models result in tissue damage in the motor cortex without concomitant motor impairment as assessed by beam crossing [42], rotarod [21,30,32,40,43,59–61], or gait analysis [40]. Others have described transient motor impairment that resolves within a week after injury [16,41,61] or between 1wk and 6mo [30,43]. Our study is unique in documenting early motor deficits in beam walking, which persisted out to 10wks after injury. Increasing the interval between injuries from 24h to 48h did not significantly reduce impairment in beam walking despite decreasing acute pathology [23], suggesting motor dysfunction after repeated CHI is not directly dependent on acute regional neuron loss or inflammation. Without a parallel group of mice with a single CHI it is not possible to completely rule out that the motor deficit was a consequence of *repeated* versus *single* mild TBI. However, assessments of beam walking 2h and 24h after the initial CHI from our previous study (not previously reported) show that one impact produces only a very slight and transient impairment which resolves by 24h, suggesting the long-lasting motor dysfunction observed in the current study is primarily due to repeated mild TBIs. Detection of longer lasting motor deficits in our model of repeated mild TBI as compared to others may be due, in part, to differences in the location, number or severity of our impacts, or to greater sensitivity of our motor task.

In addition to mild deficits in beam walking ability, mice with repeated mild TBI exhibited chronic neurodegeneration within the pyramidal tract and cerebellum. Although the pyramidal tract contains the upper motor neurons of the corticospinal tract that control voluntary movements, the amount of persistent neurodegeneration in the pyramidal tract did not correlate with beam walking deficits. It is possible that the peak of neurodegeneration in this region occurred much earlier, given that other studies of TBI have noted maximal silver staining at 48h after a severe, focal TBI [62] or 72h after a milder impact acceleration injury [63]. Therefore, further temporal studies are necessary to elucidate whether acute neurodegeneration in the pyramidal tract better predicts beam walking deficits.

Neurodegeneration in the pyramidal tract and cerebellum was coupled with astrocytosis and microgliosis at 10wks post-injury. The relationship between ongoing neurodegeneration and chronic inflammation is still debated [64,65]. However, in the current study, quantification of reactive astrocytes and activated microglia in the pyramidal tract failed to reveal a correlation with silver stain accumulation. Interestingly, increased astrocytosis correlated with improved beam walking ability, suggesting that the long-term astrocyte response may be involved in promoting recovery after injury as has been reported in experiments of spinal cord injury [66,67]. Reactive microgliosis, in contrast, appeared to be higher in mice with greater motor dysfunction. Such an inverse relationship between the activation of astrocytes and of microglia has been reported in mouse models of Alzheimer’s disease [68] and Batten disease [69]. When reactive astrocytosis was inhibited in either of these models, microgliosis was upregulated. Future studies are needed to better understand the factors that modulate the relative astrocyte and microglial responses to brain trauma.

### Memory Circuit Damage

Difficulties with memory, such as amnesia to the traumatic event or trouble with memory retention, can occur following mild TBI. Based on our previous observation of damage in the entorhinal cortex and hippocampus, regions involved in the memory circuit, we postulated that repeated CHI may result in cognitive deficits. Indeed, mice with repeated CHI had significantly lower memory scores in an NOR task across a 10wk period compared sham mice, adding to the growing literature documenting memory impairment after repeated mild TBI [21,22,34,35,38,42,48]. Future studies could evaluate the magnitude or duration of cognitive dysfunction as a function of the number of injuries to expand upon findings in previous studies [22,34,42,48]. The length of the window of vulnerability for repeated TBI may also be affected by the number of injuries. For example, an inter-injury interval of 1wk between two head injuries was sufficient to eliminate acute cognitive deficits [21], while five injuries given at 1wk intervals resulted in cognitive dysfunction that persisted between six months and a year [22,38]. Therefore, in our study of five impacts, a longer post-injury evaluation period may have helped to discern differences in the persistence of cognitive dysfunction between rCHI repeated at 24h versus 48h inter-injury intervals.

The entorhinal cortex and hippocampus exhibited little evidence of ongoing neurodegeneration, astrogliosis or microgliosis. While it is possible that an acute transient wave of neuron death and/or inflammation in these regions contributed to persistent memory dysfunction, it seems unlikely to be the major determinant since the rCHI-48h group, which had less neuron loss and microglial activation than the rCHI-24h group early after injury [23], had comparable cognitive deficits. An alternative explanation for persistent neurobehavioral impairment may be ongoing dysfunction of surviving neurons due to, for example, impairments in axonal conduction velocity or long-term potentiation. Such changes have been measured in hippocampal neurons after a blast injury which resulted in deficits in hippocampal-dependent learning and memory without macroscopic tissue damage [70]. Alternatively, metabolic measures gleaned through imaging approaches or autoradiography could reveal areas of sublethal neuron injury [28,33].

### Visual System Damage

Humans with mild TBIs often complain of disruptions in normal vision which can manifest in the form of saccades and difficulties with pursuit, convergence, accommodation and the vestibular-ocular reflex [71]. These changes can result in difficulties with reading, light sensitivity, and headaches leading to poor quality of life. Despite the high occurrence, visual dysfunction after TBI is an underrepresented area of study. Models of midline, diffuse head injury are a valuable tool for exploring visual pathway damage after mild TBI. Repeated mild CHI resulted in axonal degeneration in the optic tract and optic nerve axon loss at 10wks after injury, which was comparable for 24h and 48h inter-injury intervals. Our findings expand upon reports of neurodegeneration in the optic tract, superior colliculus and optic nerve within the first week after repeated mild head injury in mice [72] and corroborate the work of Xu et al. [72] who showed a decrease in optic nerve axon number at 10wks after repeated impact acceleration brain injuries. Both early optic tract degeneration and chronic optic nerve axon loss were greater after repeated TBI than single injury [72]. While these data suggest repeated injury causes additive damage within the visual system, our study is the first to examine visual pathway pathology with respect to inter-injury interval.

Astrocytosis and microglial activation accompanied optic tract and optic nerve neurodegeneration one week after four impact acceleration injuries over a one-week period [72]. Retinal microglial activation was also observed at 1wk after multiple injuries but did not persist to 10wks. Chronic inflammation coincided with demyelination within the optic nerve at 10 and 13 weeks after five CHI repeated at 48h intervals [45], consistent with our findings of long-term microgliosis and astrocytosis within the optic tract and optic nerve after five CHI. Analysis of CD68 in the optic tract of our archival tissue taken at 24h post-injury [23] demonstrated that a single CHI initiates a delayed transient microglial activation in the optic tract while repeated CHI led to optic tract microgliosis sustained up to 10wks post-injury. Inflammation in the optic tract was not diminished acutely or chronically by extending the inter-injury interval from 24h to 48h suggesting that the visual pathway is more susceptible to CHI than the entorhinal cortex and hippocampus. Increased susceptibility for damage in the visual system may be due to the optic nerve’s location beneath and its separation from the cerebrum. Visual system damage raises the possibility that repeated mild TBI induces visual dysfunction. Although retinal ganglion cell loss was not observed after a single mild fluid percussion injury [73], it has been reported after repeated closed head impact [45] and repeated impact acceleration [72]. Electroretinography traces in mice after repeated CHI showed a decreased photopic negative response compared to that in sham animals [45], suggestive of retinal ganglion cell dysfunction [74] which could influence performance in vision-based tasks such as the NOR task. However, mice with visual pathway neurodegeneration following repeated impact acceleration did not perform poorly in a visible platform trial suggesting the damage may not induce substantial vision impairment [75]. The NOR task used in this study incorporates large objects with marked differences in their shapes and sizes. Rodents rely heavily on their whisker sensation and olfaction more so than their vision when exploring their surroundings, lessening the possible confound of diminished vision. However, further vision function tests are warranted to better understand potential consequences of visual pathway damage after mTBI.

### Conclusion

Several reports have concluded that post-concussive symptoms in humans do not correlate with the presence or absence of positive neuroimaging findings acutely after injury [76–78]. In conjunction with our previous work we provide evidence for a similar phenomenon in mice. Despite significant differences in acute histopathology following five mild TBIs repeated at 24h or 48h intervals, repeated CHI resulted in persistent deficits in beam walking and novel object recognition in mice up to 10wks after injury that were not significantly reduced by extending the inter-injury interval. Neuronal degeneration and gliosis observed acutely in the entorhinal cortex and hippocampus did not persist out to 10wks after injury. Tau pathology was not observed after repeated mild TBI, adding to a growing number of mild TBI studies in wildtype mice that report limited or no tau-positive immunopathology (see review by Ojo et al. [79]). However, axonal degeneration and inflammation in the optic tract, optic nerve, pyramidal tract and cerebellum were notable even 10wks after the final injury. Additional work with longer post-injury evaluations, longer periods of rest between injuries, as well as with single head injuries would help determine if further increasing the interval between injuries can reduce the chronic consequences of mild TBI.

## Supporting Information

S1 Fig Hemotoxylin and Eosin stain following repeated closed head injury (rCHI) or repeated sham injury (rSHAM). No overt cell loss was observed in the cerebrum (top panel) or cerebellum (bottom panel) of mice after rCHI at 24h or 48h inter-injury intervals compared to mice that received rSHAM injury.
(TIF)

S2 Fig Acute microgliosis in the optic tract after single and repeated closed head injuries (CHI). The percent area of immunohistochemical labeling of cluster of differentiation 68 (CD68) was quantified in the optic tract of mice receiving repeated sham injury (rSHAM), single CHI (euthanized at 24h, 5d, and 9d after injury) and five repeated CHI at 24h or48h inter-injury intervals (euthanized 24h after the final injury) for comparative analysis. $ indicates p<0.05 compared to sham.! indicates p<0.05 compared to (single) CHI 24h. # indicates p<0.05 compared to CHI 5d.
(TIF)

S3 Fig PHF-1 after repeated closed head injury (CHI) compared to positive control tissue. Hippocampal image from 3.5mo old rTg4510 tau mouse immunohistochemically labeled with Paired Helical Filament 1 (PHF-1; A). Black arrowheads indicate positive tau inclusions. Repeated CHI did not induce PHF-1 positive tau inclusions by 10wks after injury (B). Scale bar: 125μm.
(TIF)
