# Supplementary figures and images for: Repeated Closed Head Injury in Mice Results in Sustained Motor and Memory Deficits and Chronic Cellular Changes
Source: PLoS One. 2016 Jul 18;11(7):e0159442. doi: 10.1371/journal.pone.0159442 (PMC4948770; doi:10.1371/journal.pone.0159442)

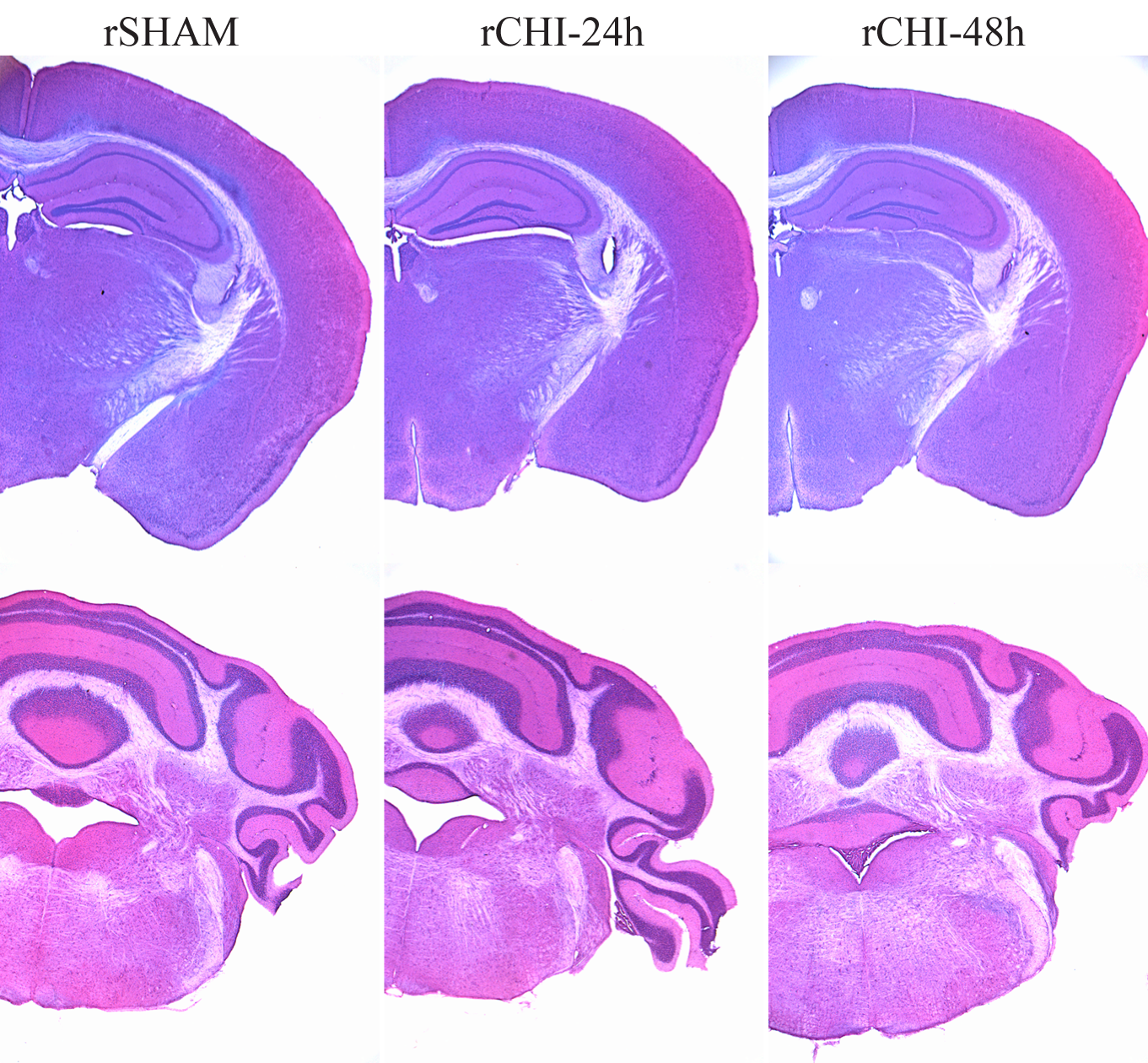

Supplement: S1 Fig — No overt cell loss was observed in the cerebrum (top panel) or cerebellum (bottom panel) of mice after rCHI at 24h or 48h inter-injury intervals compared to mice that received rSHAM injury. (TIF) [file pone.0159442.s001.tif]

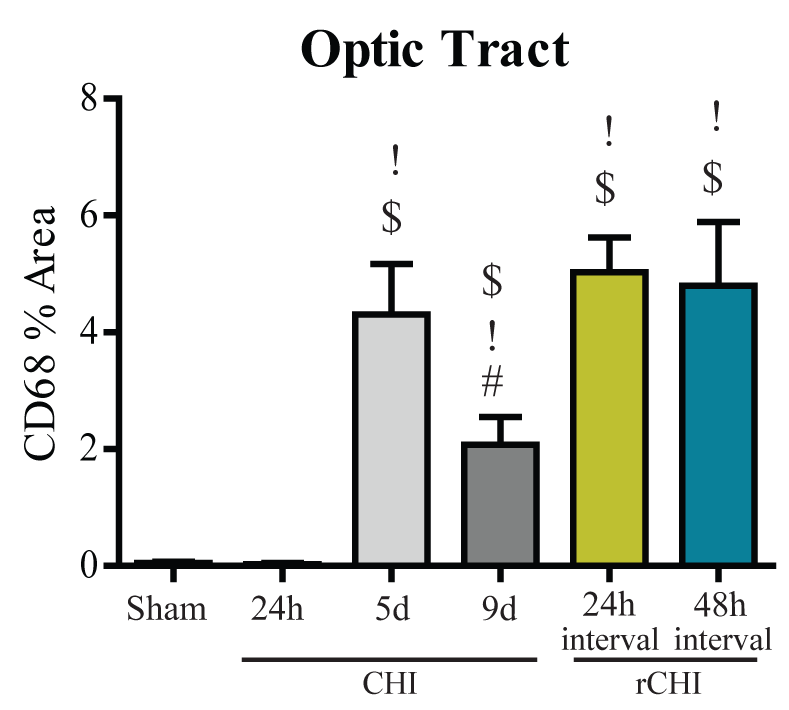

Supplement: S2 Fig — The percent area of immunohistochemical labeling of cluster of differentiation 68 (CD68) was quantified in the optic tract of mice receiving repeated sham injury (rSHAM), single CHI (euthanized at 24h, 5d, and 9d after injury) and five repeated CHI at 24h or48h inter-injury intervals (euthanized 24h after the final injury) for comparative analysis. $ indicates p<0.05 compared to sham.! indicates p<0.05 compared to (single) CHI 24h. # indicates p<0.05 compared to CHI 5d. (TIF) [file pone.0159442.s002.tif]

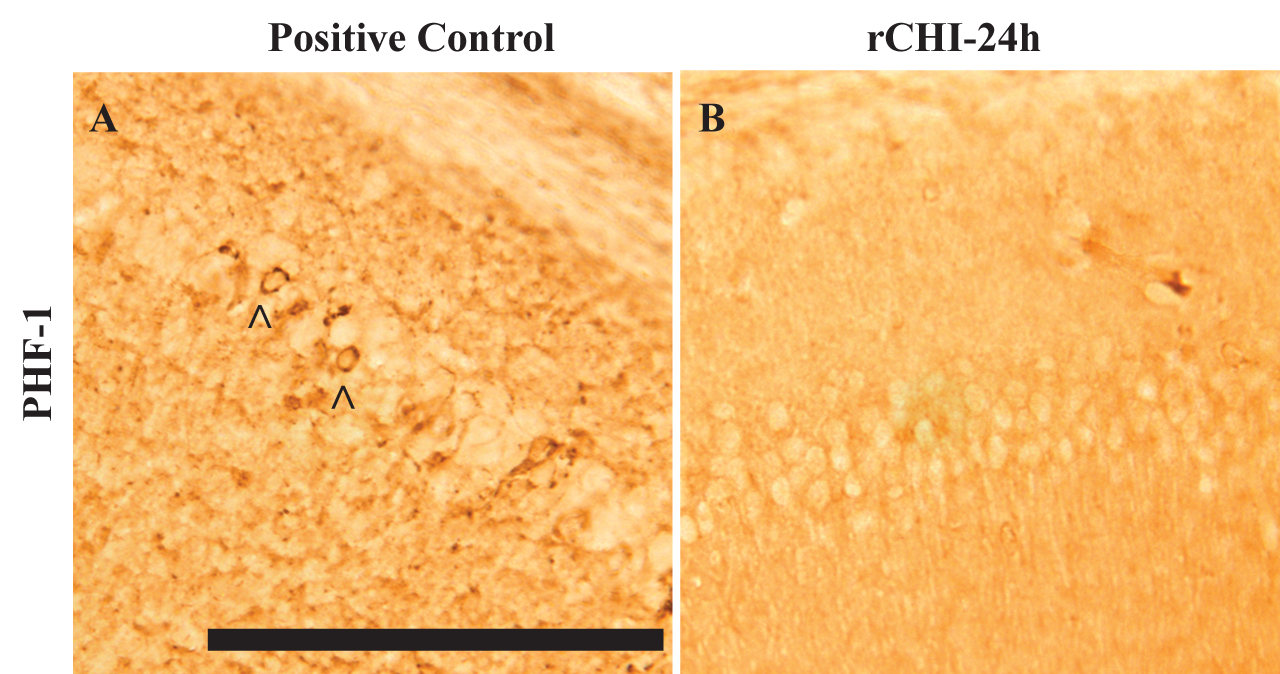

Supplement: S3 Fig — Hippocampal image from 3.5mo old rTg4510 tau mouse immunohistochemically labeled with Paired Helical Filament 1 (PHF-1; A). Black arrowheads indicate positive tau inclusions. Repeated CHI did not induce PHF-1 positive tau inclusions by 10wks after injury (B). Scale bar: 125μm. (TIF) [file pone.0159442.s003.tif]
